# Targeting female flight for genetic control of mosquitoes

**DOI:** 10.1371/journal.pntd.0008876

**Published:** 2020-12-03

**Authors:** David Navarro-Payá, Ilona Flis, Michelle A. E. Anderson, Philippa Hawes, Ming Li, Omar S. Akbari, Sanjay Basu, Luke Alphey

**Affiliations:** 1 Arthropod Genetics, The Pirbright Institute, Pirbright, United Kingdom; 2 Bioimaging, The Pirbright Institute, Pirbright, United Kingdom; 3 Cell and Developmental Biology Section, Division of Biological Sciences, University of California, San Diego, California, United States of America; University of Queensland, AUSTRALIA

## Abstract

*Aedes aegypti Act4* is a paralog of the *Drosophila melanogaster* indirect flight muscle actin gene *Act88F*. *Act88F* has been shown to be haploinsufficient for flight in both males and females (amorphic mutants are dominant). Whereas *Act88F* is expressed in indirect flight muscles of both males and females, expression of *Act4* is substantially female-specific. We therefore used CRISPR/Cas9 and homology directed repair to examine the phenotype of *Act4* mutants in two Culicine mosquitoes, *Aedes aegypti* and *Culex quinquefasciatus*. A screen for dominant female-flightless mutants in *Cx*. *quinquefasciatus* identified one such mutant associated with a six base pair deletion in the *CxAct4* coding region. A similar screen in *Ae*. *aegypti* identified no dominant mutants. Disruption of the *AeAct4* gene by homology-dependent insertion of a fluorescent protein marker cassette gave a recessive female-flightless phenotype in *Ae*. *aegypti*. Reproducing the six-base deletion from *Cx*. *quinquefasciatus* in *Ae*. *aegypti* using oligo-directed mutagenesis generated dominant female-flightless mutants and identified additional dominant female-flightless mutants with other in-frame insertions or deletions. Our data indicate that loss of function mutations in the *AeAct4* gene are recessive but that short in-frame deletions produce dominant-negative versions of the AeAct4 protein that interfere with flight muscle function. This makes *Act4* an interesting candidate for genetic control methods, particularly population-suppression gene drives targeting female viability/fertility.

## Author summary

The yellow fever mosquito and the Southern house mosquito are important vectors of infectious diseases. Given their widespread presence across tropical and subtropical regions of the world and the increased risk of spread due to global warming there is a growing need for population control. Gene drives aim to spread a genetic element within target genes required for mosquito reproduction to disrupt their function and crash a population. Female-specific genes provide interesting candidates for population control since female mosquitoes determine the reproductive capacity of a population as well as being the actual vectors of disease. Here we describe a study on *Actin-4* loss in both *Aedes aegypti* and *Culex quinquefasciatus*, where we observe female-specific disruption of flight ability and propose it as a candidate for genetic methods of population suppression.

## Introduction

Insect-borne diseases have a huge impact on human health worldwide, in particular mosquito-borne diseases which result in over 1 million deaths every year. Around 17% of the world’s infectious disease disability adjusted life year (DALY) burden is estimated to be due to insect-borne diseases, of which 90% is attributed to mosquito-transmitted diseases [1].

With the advent of promising gene drive strategies, such as homing endonuclease based or CRISPR-Cas9 daisy chain drives [2–4], there is a growing need to study potential target genes in key target mosquito species such as the yellow fever mosquito, *Aedes aegypti* (L.), and the Southern house mosquito, *Culex quinquefasciatus* (Say). *Ae*. *aegypti* is an anthropophilic mosquito present in Central and South America, Africa, South East Asia, and Oceania [5]. It is the principal vector of human dengue which has an estimated incidence of 390 million infections per year worldwide [6]. It is also an important vector of other arbovirus diseases such as yellow fever [7], Chikungunya [8] and Zika [9]. *Cx*. *quinquefasciatus* is a widespread mosquito found in tropical and subtropical regions of the world and is a vector of both human and wildlife pathogens [10]. This species is the main vector of the avian malaria parasite *Plasmodium relictum* [11,12], as well as the human parasitic worm (*Wuchereria bancrofti*) [13,14] and is also able to transmit arboviruses such as West Nile virus [15,16].

A leading design of gene drive for population suppression has a ‘homing’ drive located in and disrupting a target gene resulting in a corresponding increase between frequency of both the gene drive and the loss-of-function alleles [2,3,17]. If the target gene is required for viability or fertility, this will lead to population suppression. Reduced ability of the gene drive to spread can be minimized by employing a recessive mutant phenotype, e.g. only females, and if the target gene is required only in somatic cells while homing is restricted to germline cells. Other designs require dominant lethal phenotypes, again preferably female-specific [18]. Few genes affecting female viability/fertility are known in mosquitoes, though analysis of ovarian expression identified some candidates in *Anopheles gambiae* [19]. We investigated a different functional process, flight, as a potential source of target genes. *Ae*. *aegypti* mate in flight, and the female wingbeat frequency is a primary mating recognition cue, therefore flightless females are functionally sterile [20,21] as well as being unable to disperse from their larval pools, evade predators or blood feed, let alone a second time ensuring lack of transmission.

Mechanical power during insect flight is provided by the action of indirect flight muscles (IFMs) in the adult thorax [22]. Actin filaments in these fibrillar muscles are formed by polymerization of a particular actin isoform; Actin-3 in *Drosophila melanogaster* [23] encoded by *Act88F* [24]. *Actin-4*, present in both *Ae*. *aegypti* and *Cx*. *quinquefasciatus*, is a paralog of *Act88F* but with actin-coding transcripts expressed in IFMs of females only [25,26]. The promoter of *AeAct4* has been previously exploited to develop a RIDL strain displaying a repressible female flightless phenotype [27]. The same strategy has been shown to work in *Aedes albopictus*, and also that *Act4* promoters from either species are functional in the other species [25].

Classical mutagenesis studies indicated that *Act88F* is haploinsufficient for flight in both male and female *D*. *melanogaster* adults [23,28]. Haploinsufficient genes are rare in nature; apart from *Act88F*, the few examples known in the model organism *D*. *melanogaster* are primarily ribosomal protein genes [29]. Both haplosufficient and haploinsufficient genes for female viability or fertility are potentially useful for genetic control. Haplosufficient genes would make useful target genes in a daisy chain drive configuration whilst haploinsufficient genes could be part of an underdominance based drive [4,30]. We therefore investigated the phenotype of mutations in *AeAct4* (AAEL001951) in *Ae*. *aegypti* and *CxAct4* (CPIJ012572) in *Cx*. *quinquefasciatus*.

## Methods

### Mosquito rearing

The *Ae*. *aegypti* Liverpool (LVP) strain originated from West Africa and has been maintained at the Liverpool School of Tropical Medicine since 1936 [31]. The *Cx*. *quinquefasciatus* Pel strain was originally established from larvae collected in Peliyagoda, Sri Lanka in 1984 [32]. An *exu-Cas9 Ae*. *aegypti* line [33] was used in a set of microinjections due to its Cas9 germline activity. The *exu-Cas9* line was highly enriched through the removal of any marker negatives for at least 3 generations.

Larvae were reared on dry fish food (TetraMin, Tetra GmbH, Germany) and adults were provided 10% sucrose *ad libitum*. Insectary rearing conditions were maintained at 27°C (± 2°C), 70% (± 10%) relative humidity and 12:12 light/dark cycle. For egg collection, adult females were fed on defibrinated horse blood (TCS Biosciences Ltd, Buckingham, UK) at 37°C using a Hemotek membrane feeding system (Hemotek Ltd, Blackburn, UK).

### Microinjection

Microinjection into *Ae*. *aegypti* and *Cx*. *quinquefasciatus* pre-blastoderm embryos was carried out following published protocols [34,35]. Clustered regularly interspaced short palindromic repeats (CRISPR)/Cas9 homology directed repair (HDR) injections were carried out as described [36], except injected G_0_ males were crossed instead of discarded. Cas9 microinjections used recombinant Cas9 protein, which was obtained commercially (PNA Bio, Newbury Park, CA, US). Injection mixes consisted of 40 ng/μl of each synthetic single guide RNA (sgRNA), 400 ng/μl of Cas9 protein, 700 ng/μl of dsDNA HDR donor plasmid, and injection buffer [37]. Male G_0_ adults were individually crossed to 5 females in 12 ounce clear plastic deli pots (Ambican, London, UK) and then pooled in groups of 20 G_0_ males per cage for blood-feeding and egg collection. Female G_0_ adults were crossed to males in a 1:1 ratio in pools of 100 G_0_ females per cage for blood-feeding and egg collection. 15x15x15cm Bugdorm cages (NHBS Ltd, Devon, UK) were used for pooling individuals after being crossed. G_1_ larvae were screened for mCherry fluorescence using a Leica MZ10 microscope (Leica Biosystems, Wetzlar, Germany). Transgenic lines were established from single G_1_ positives. The *exu-Cas9* line was used for one set of microinjections in *Ae*. *aegypti* and did not require the described co-injection of recombinant Cas9 protein. Considering that *exu-Cas9* adults were not all homozygotes, eggs collected for microinjection were obtained from individual females identified as expressing the fluorescent marker.

### sgRNA design and plasmid construction

The *Ae*. *aegypti* Actin-4 gene sequence, AAEL001951, has been previously identified [26] and CPIJ012572 is annotated as a one-to-one ortholog in Vectorbase [38].

CRISPR/Cas9 sgRNAs were designed using CHOP-CHOP online prediction tool [39] to target approximately the first 200 bp of the coding region to maximize the likelihood of generating null alleles. Candidate sgRNAs (S1 Table) were assessed for *in vitro* efficacy through cutting of an *Act4* template by Cas9 protein (S2 Table). LA1429, the single stranded oligo DNA nucleotides (ssODN) donor for HDR, was designed following standard protocols for HDR in *Ae*. *aegypti* [36].

AGG1070, the *AeAct4* HDR 3xP3-mCherry dsDNA donor, was built through three-way Golden Gate cloning [40] of the 3xP3-mCherry-SV40 marker cassette (amplified from an existing plasmid, AGG1069) and the two *AeAct4* homology arms (2Kb each) that were amplified from LVP genomic DNA. Primer sequences are provided in S4 Table. Plasmid sequences are provided in S5 Table.

### Analysis of CRISPR/Cas9 induced mutations

Genomic DNA was extracted from individual mosquitoes using the NucleoSpin Tissue kit (Macherey-Nagel, Düren, Germany). The putative mutated region was PCR amplified to detect CRISPR/Cas9 mediated indels or HDR integrations using primers shown in S3 Table. Selected amplicons were Sanger sequenced (Eurofins, Ebersberg, Germany) and the chromatograms were analyzed to determine the nature of the mutations.

### Flightless phenotype analysis

G_1_ adults were assessed for flight ability in 15x15x15cm Bugdorm cages (NHBS Ltd, Devon, UK). A sucrose source (10% sucrose-soaked cotton wool) was placed at the bottom of cages to be accessible without requiring flight ability. Ability to fly was assayed by flight, or lack thereof, upon tapping the cage as previously described in mosquitoes [25,27]. Initial selection of flightless individuals was not stringent, bad fliers and individuals with a range of wing or other defects were isolated. These were considered ‘flightless’ in the final count if they were fully flightless for ~7 days. Nonetheless, all individuals from the initial selection were also analyzed molecularly.

### Transmission electron microscopy

*AeAct4*^hdr1^ females (heterozygous) were crossed to *AeAct4*^hdr1^ males in order to obtain double knock-out and thus non-flying females. Legs, wings, heads and abdomens were removed from 5–7 day-post-emergence adult females and individual thoraces were prepared for TEM. Thoraces were placed in 2% glutaraldehyde in phosphate buffer for 3 hours, after which time the fixative was replaced with 1% aqueous osmium tetroxide. After 90 minutes samples were dehydrated in a series of ethanol dilutions as follows; 70% ethanol for 45 minutes, 90% ethanol for 30 minutes, 3 x 100% ethanol for 15 minutes each. After a 15 minute wash in propylene oxide, samples were placed in a 1:1 mix of propylene oxide and freshly made Agar 100 epoxy resin for 60 minutes before a 2 hour infiltration period in pure epoxy resin. Thoraces were mounted in flat embedding molds before being placed in the polymerization oven at 60°C for 16–24 hours. Every step before polymerization was carried out at room temperature on a rotator to agitate the samples in the solutions. Glutaraldehyde, osmium tetroxide, propylene oxide and Agar 100 epoxy resin were all sourced from Agar Scientific Ltd, UK. Thick sections (0.5μm) were cut using a Leica UC7 ultramicrotome, stained with aqueous 1% toluidine blue and viewed in a transmission light microscope (Leica Microsystems Ltd.) to identify areas of indirect flight muscle. Thin sections (70 nm) of the correct areas were cut, collected on 200 mesh thin bar copper grids and stained with uranyl acetate and lead citrate using a Leica EM Stain before being imaged in a FEI Tecnai12 TEM with a Tietz F214 CCD camera or Thermo Fisher Talos L120C TEM with Ceta camera.

### Statistical analysis

The Chi-squared test was conducted using Microsoft Excel to determine the statistical significance between observed and expected progeny distributions.

## Results

### Generation of flightless phenotype in *Cx*. *quinquefasciatus*

Recent advances in the use of CRISPR/Cas9 mutagenesis in *Ae*. *aegypti* have facilitated genetic analysis of individual genes of interest in non-model organisms including mosquitoes [33,36]. *In vivo* mutagenesis of *Act4* in *Cx*. *quinquefasciatus* (*CxAct4*) was carried out by co-injecting 915 G_0_ eggs with sgRNAs 1–4 and Cas9 protein (Table 1). G_0_ survivors were outcrossed individually to wild-type (Pel) adults to be able to observe potential mutations in a heterozygous genetic background. All G_0_ adults were able to fly normally, showing no evident phenotype despite their presumed mosaic nature. Despite the low G_0_ survival rate (0.7%) and the few G_1_ adults obtained (8 males and 8 females, from a single egg raft from one G_0_ founder) compared to published rates [34,41,42], 3 flightless females were identified. Sanger sequencing of *CxAct4* in flightless G_1_ females indicated these flightless females were heterozygous for a 6bp deletion (TGCCTA) of nucleotides 156 through 161 from the start codon, with the A of the ATG being 1 (156_161delTGCCTA). Interestingly, analysis of *CxAct4* in 5 flying males revealed the same in-frame deletion in 1 individual. The identified mutation affects three different codons, resulting in the deletion of two amino acids (A53_Y54del), as well as use of an alternative codon (GAC instead of GAT) for amino acid D52. This mutation appears to be dominant for lack of flight in *Cx*. *quinquefasciatus* females.

**Table 1:** CRISPR Cas9/sgRNA *Act4* mutagenesis in *Cx*. *quinquefasciatus* and *Ae*. *aegypti*.

Mosquito species (strain)	Injected components	Injected G_0_ eggs	Adult G_0_ survivors (%)	G_0_ mosaic females (%)	G_0_ founders (%)	G_1_ flightless female adults/total female G_1_ (%)	G_1_ sequence confirmed *Act4* mutant flightless females (%)	Mutation Identified
*Cx*. *quinquefasciatus* (Pel)	*CxAct4* sgRNAs (1–4), Cas9 protein	915	6 (0.7)	0 (0)	1 (17)	3/8 (38)	3/3 (100)	156_161delTGCCTA (A53_Y54del)
*Ae*. *aegypti* (Liverpool)	*AeAct4* sgRNAs (1, 2), Cas9 protein	1802	67 (4)	0 (0)	0 (0)	1/3713 (0.03)	0/1 (0)	-
*Ae*. *aegypti* (Liverpool)	*AeAct4* sgRNAs (3, 4), Cas9 protein	736	68 (9)	0 (0)	0 (0)	1/3422 (0.03)	0/1 (0)	-

*Act4* CRISPR/Cas9 mutagenesis in 2 mosquito species. Potential mutants were screened using a flight assay. sgRNA concentration was 40 ng/μl and Cas9 protein 300 ng/μl.

An equivalent *Act4 in vivo* mutagenesis attempt was carried out in *Ae*. *aegypti* by co-injecting embryos with sgRNAs and Cas9 protein. All injection survivor G_0_ adults were able to fly normally, showing no evidence of a mosaic effect. One flightless G_1_ female was isolated from each set of injections (Table 1). However, following analysis of Sanger sequencing of the *AeAct4* locus no mutation was identified.

### *AeAct4* gene disruption leads to recessive female-specific loss of flight ability

**Fig 1:**
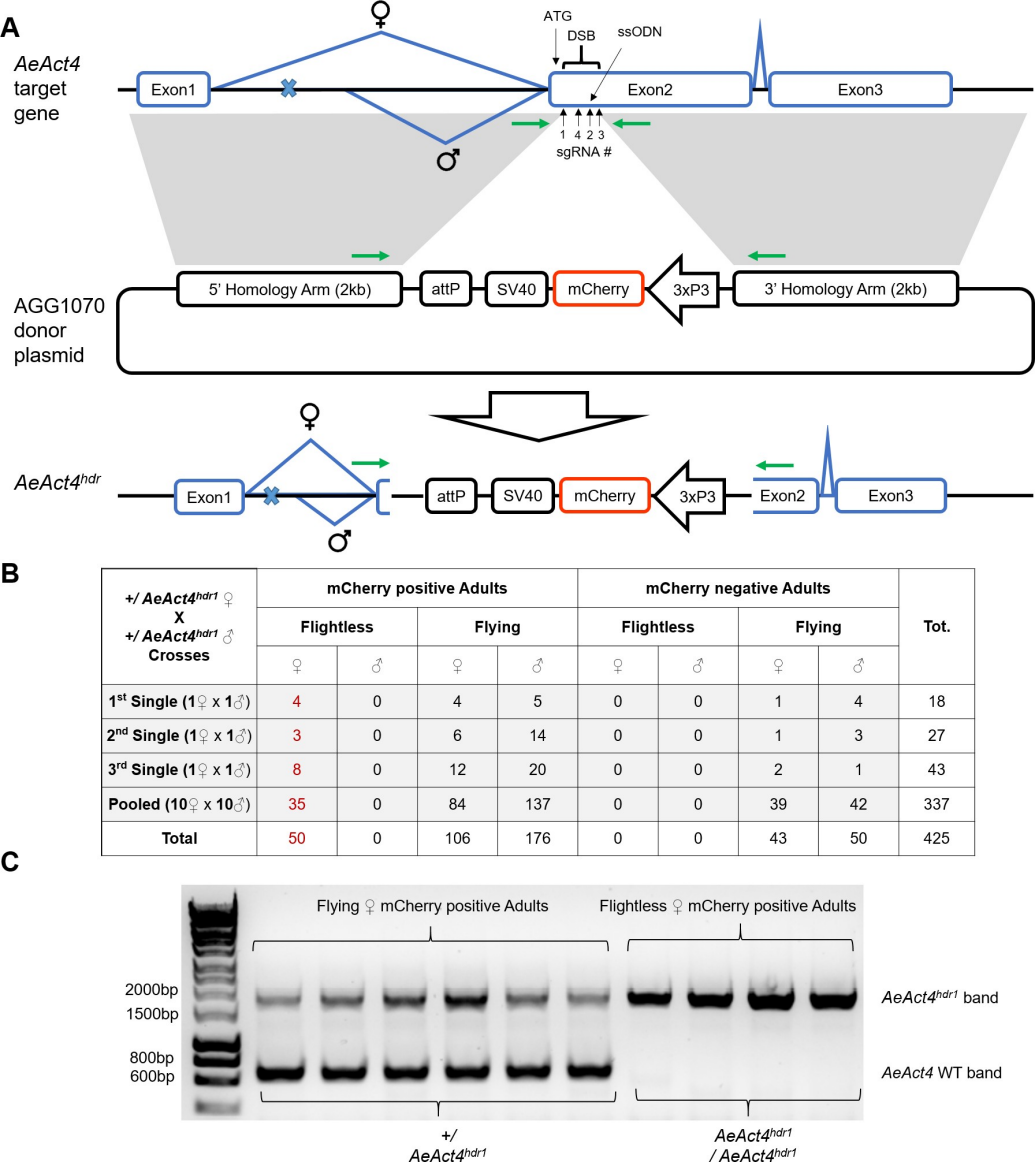
*AeAct4* gene disruption by homology-directed repair results in female-specific recessive lack of flight ability. (**A**) Injected Cas9 protein and sgRNAs targeting *AeAct4* induce a double stranded break (DSB) at the target site. A donor plasmid (AGG1070) having 2kb homology arms corresponding to the immediate upstream and downstream regions of the outermost cut sites was injected as a template for HDR. A 3xP3-mCherry-SV40 cassette serves as a marker for integration. Male-specific alternative splicing incorporates a number of early start and stop codons (marked by blue x) which ablate AeAct4 translation. Green arrows represent primer pair used for amplicons shown in (c). (**B**) 50 flightless females were identified out of a total of 156 marker-positive female progeny (32%). (**C**) Molecular confirmation of 6 flying and 4 flightless mCherry positive female adults, individually analyzed by PCR using the primers indicated in (A). *AeAct4*^hdr1^ and WT amplicons were generated. Based on this PCR assay, of the marker-positive females analyzed, all fliers were heterozygous for *AeAct4*^hdr1^ and all flightless females were homozygous.

Disruption of *Act4* in *Ae*. *aegypti* was achieved via insertion of a marker cassette through homology directed repair (HDR) (Fig 1A). The HDR donor construct (AGG1070) consisted of a *3xP3-mCherry-SV40* marker cassette flanked by 2kb homology arms corresponding to the immediate upstream and downstream regions of the outermost *AeAct4* sgRNA cut sites [43]. *AeAct4* sgRNAs targeted the first 220 nucleotides of the coding region to increase likelihood of generating non-functional protein. 600 G_0_ eggs were co-injected with a donor plasmid (AGG1070), sgRNAs (1–4), and Cas9 protein (Table 2). All G_0_ adults were able to fly normally, showing no evidence of a mosaic effect. G_1_ larvae were screened, for presence of the fluorescent marker. Both marker positive and negative female G_1_ adults were analyzed for their flight ability.

**Table 2:** HDR mediated CRISPR Cas9/sgRNA *AeAct4* mutagenesis in *Ae*. *aegypti*.

				Knock-out only (no HDR marker)			HDR mediated knock-in	
Injected components	Injected G_0_ eggs	Adult G_0_ survivors (%)	G_0_ mosaic females (%)	G_0_ founder pools	flightless G_1_ females /total G_1_ females (%)	G_1_ *AeAct4* mutant flightless females (%)	G_0_ founder pools	G_1_ marker positive adults/total G_1_ (%)
AGG1070 *AeAct4* donor plasmid, Cas9 protein, *AeAct4* sgRNAs (1–4)	600	97 (16.2)	0 (0)	5	8/3557 (0.2)	2/8 (25)	1	14/7198 (0.2)

*AeAct4* inactivation by CRISPR/Cas9 mediated knock-in of a marker cassette. An initial fluorescence marker screen identified knock-ins from WT or knock-outs. sgRNA concentration was 40 ng/μl, Cas9 protein 400 ng/μl and donor plasmid 700 ng/μl.

14 G_1_ larvae were identified by expression of the mCherry marker. The resulting transgenic line was named *AeAct4*^hdr1^. All the *AeAct4*^hdr1^ marker positive G_1_ adults were able to fly normally. Transgene heterozygotes were interbred and their progeny analyzed for flight ability (Fig 1B). All male progeny were able to fly normally, however a total of 50 out of 156 marker-positive females were identified as flightless. This is not significantly different from the 1:2:1 ratio expected in females (WT:*AeAct4*^*hdr1*^ heterozygotes:*AeAct4*^*hdr1*^ homozygotes) from the hypothesis that all genotypes are viable but homozygous females cannot fly, i.e. that *AeAct4* disruption induces a recessive female flightless phenotype (X^2^ = 1.34, d.f. = 2, p = 0.51). The genetic integration into *AeAct4* gene was confirmed by sequence analysis of PCR products generated from *AeAct4*^hdr1^ individuals: 4 flying and 6 flightless female G_2_ adults (Fig 1C). PCR across the insertion gave two amplicons (WT and *AeAct4*^hdr1^ allele) in all flying G_2_ individuals (heterozygotes) whilst only the *AeAct4*^hdr1^ amplicon appears in flightless G_2_s (homozygotes). The phenotype of this insertion, and probably the null phenotype of *AeAct4*, is therefore recessive female-specific loss of flight ability.

Additionally, from the marker negative G_1_ adults, 8 flightless females were identified, of which 2 carried mutations (34_40delGACAACG and 207_209delATA) in the *AeAct4* locus. The two confirmed mutations in non-marker flightless females appear to be generated by sgRNA 1 and sgRNA 3 respectively. The 6 flightless females which do not show mutations in *AeAct4* could correspond to the rate of flightless adults observed in WT samples. The other 2 flightless females carried different mutations which may or may not be responsible for the flightless phenotype. The 207_209delATA mutant could be a dominant negative version since it is in frame whilst 34_40delGACAACG is an out of frame 7bp deletion and is unlikely to result in a dominant negative.

### The 6bp in-frame deletion generated in *Cx*. *quinquefasciatus* is dominant negative for flight in *Ae*. *aegypti* females

Since the single *CxAct4* mutant was dominant, yet amorphic mutants of *AeAct4* are recessive, we speculated that the *CxAct4* mutant might represent a dominant negative mutant. To investigate this, we conducted HDR mutagenesis of *AeAct4*, aiming to replicate the *CxAct4* deletion (156_161delTGCCTA) in *Ae*. *aegypti*. A 120bp single stranded oligonucleotide donor (ssODN) was designed (LA1422) to contain 60bp upstream and downstream of 156_161delTGCCTA but not the deleted sequence itself. 580 *Ae*. *aegypti* G_0_ eggs from an *exu-Cas9* line [33] were co-injected with *AeAct4* sgRNA2 and ssODN (Table 3). Of 187 adult G_0_ survivors (32.2% survival rate), 85 were females. After analyzing their flight ability, 5 of these G_0_ females (5.9%) were not able to fly suggesting at least a mosaic effect. The other G_0_ adults were backcrossed to wild-type (LVP) mosquitoes. G_1_ individuals were analyzed for flight ability and 27 flightless G_1_ females were identified, from 7 of the 9 G_0_ pools. The mutation rate observed when exu-Cas9 line was used for injections is notably higher than the rate observed when Cas9 protein was used (Table 2 knockout only G_1_ flightless 0.2%, and Table 3 G_1_ flightless females 1.4%), although the donor template for HDR was single stranded in this case. A sample of these G_1_ individuals (n = 17) were analyzed through Sanger sequencing. 14 of the 17 flightless females showed mutations in the *AeAct4* locus; the other 3 flightless females and all of the 17 flying females appeared to be wild-type in the sequenced region. Out of these 14 mutants, 6 individuals from 2 different pools had the expected in-frame 6bp deletion (156_161delTGCCTA). Each of the other 8 mutant G_1_ flightless females had other in-frame mutations at the sgRNA2 target site (Table 4).

**Table 3:** 156_161delTGCCTA ssODN HDR CRISPR/Cas9/sgRNA *AeAct4* mutagenesis in *Ae*. *aegypti*.

						17 flightless and 17 flying female G_1_s were processed for *AeAct4* sanger sequencing		
Injected components	No. Injected	G_0_ survivors (%)	G_0_ mosaic females (%)	G_1_ flightless female adults/total female G1s (%)	G_0_ founders (%)	156_161delTGCCTA *AeAct4* mutant flightless females/total G_1_ females (%)	*AeAct4* mutant flightless females/total flightless female G_1_s sequenced (%)	*AeAct4* mutant flying females/total flying female sequenced (%)
LA1422 ssODN *AeAct4* donor oligo, *AeAct4* sgRNA 2	580	187 (32.2)	5 (5.9)	27/1978 (1.4)	≥2 (1)	6/1978 (0.3)	14/17 (82)	0/17 (0)

The 156_161delTGCCTA 6bp deletion found in *Cx*. *quinquefasciatus* was replicated in *Ae*. *aegypti* via ssODN HDR CRISPR/Cas9 mutagenesis of an *exu*-Cas9 line [33]. Potential flightless G_0_ mosaics and G_1_ mutants. 17 flightless and 17 flying G_1_s were selected for Sanger sequencing of *AeAct4*. All flying G_1_s had wild type sequence. 14 out of the 17 selected flightless G1s had in-frame indels, 6 of which showed the intended 156_161delTGCCTA deletion. These 6 individuals originated from 2 different G_0_ pools. G_0_s were crossed in pools; hence, the minimum value for founders. To assess the rate of targeted mutation, rather than all mutation, G_0_s were only considered founders if they resulted in G_1_s with successful HDR events reproducing the donor template 6bp deletion; founders of other mutagenesis events are not included. sgRNA concentration was 40 ng/μl and donor oligo 125 ng/μl.

**Table 4:** Potential antimorphic mutations from ssODN HDR CRISPR Cas9/sgRNA *AeAct4* mutagenesis in *Ae*. *aegypti*.

*AeAct4* Mutation	Amino acids changed	Description	In frame?	G_1_ flightless females
122_157delACCAGGGTGTGATGGTCGGTATGGGTCAAAAAGATG	H41_A53delinsP (delHQGVMVGMGQKDA/insP)	36bp deletion	Yes	2A
127_162delGGTGTGATGGTCGGTATGGGTCAAAAAGATGCCTAC	G43_Y54del (delGVMVGMGQKDAY)	36bp deletion	Yes	2B
159_164delCTACGT	Y54_V55del	6bp deletion	Yes	4A, 4B, 4C
156_161delTGCCTAinsCGGCAC	A53_Y54delinsGT	6bp substitution	Yes	7A
159_161delCTA	Y54del	3bp deletion	Yes	8A
160_170delTACGTCGGTGAinsGACAATTT	Y54_D57delinsDNF (delYVGD/insDNF)	11bp substituted with 8bp	Yes	8D

Additional potentially dominant negative mutations from ssODN mutagenesis are shown. 8 flightless females had mutations other than the intended 156_161delTGCCTA deletion. G_1_ flightless females are numbered by the G_0_ pool with a different letter for each flightless individual from the same G_0_ pool.

### Loss of *Act4* in homozygous null *Ae*. *aegypti* females leads to disrupted muscle fibers in female indirect flight muscles

**Fig 2:**
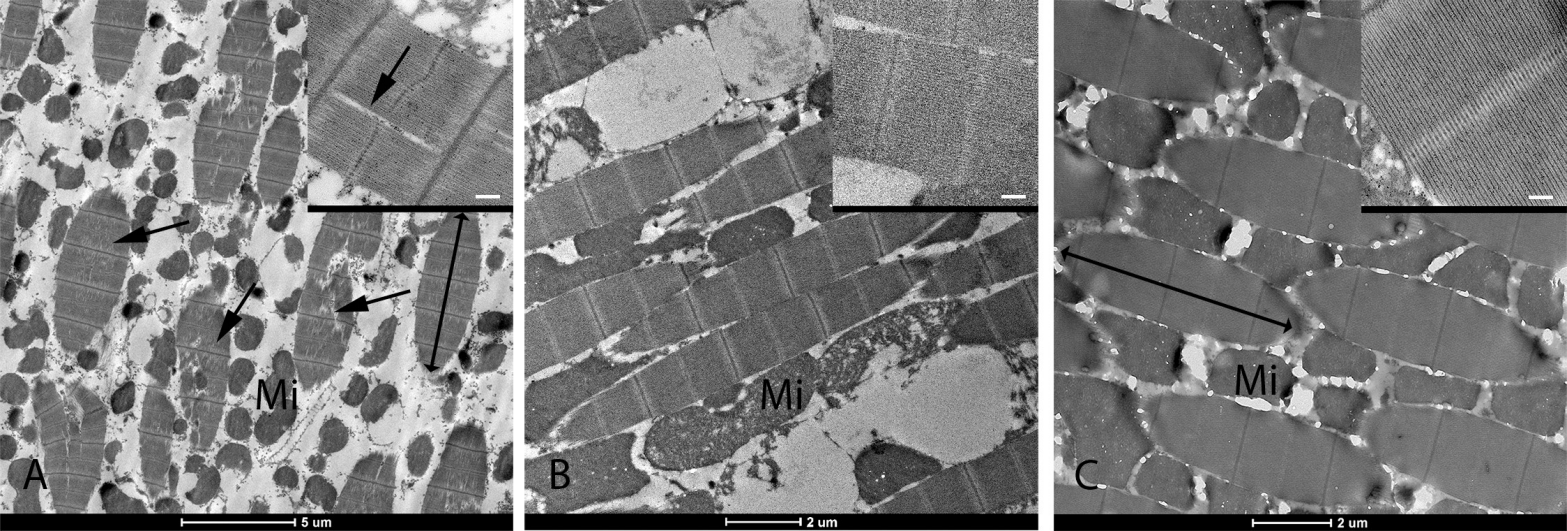
Transmission electron microscope images of the indirect flight muscles of *Ae*. *aegypti* female adults arranged within the thorax. Filaments in the myofibrils appear to be disrupted in *AeAct4*^hdr1^ homozygotes (A, arrows and inset) but not in *AeAct4*^hdr1^ heterozygotes (B, inset) or WT (C, inset). Individual myofibrils are indicated with double-headed arrows, and mitochondria (Mi) in the muscle fiber cells are clearly visible. Insert scale bars = 200 nm.

Indirect flight muscles are arranged perpendicularly within the thorax. These muscle fibers are composed of groups of largely parallel myofibrils interspersed with numerous mitochondria. Indirect flight muscles of dissected female adults (*AeAct4*^hdr1^ homozygotes, n = 3 and *AeAct4*^hdr1^ heterozygotes, n = 1) as well as LVP adults (n = 2) were imaged using transmission electron microscopy (TEM, Fig 2).

The myofibrils in homozygotes (Fig 2A) do not display the normal regular pattern of tightly bundled actin and myosin filaments running parallel and close to each other. Overall, the filaments show a more disrupted and irregular arrangement, when compared to those of the heterozygotes (Fig 2B) or WT (Fig 2C).

## Discussion

We have shown that disruption of the *Act4* gene by knock-in of a large insertion leads to a recessive, female-specific, loss-of-flight phenotype, which we propose is the amorphic phenotype of this gene. This notably differs from the phenotype of the *Drosophila* orthologue *Act88F* by being recessive and female specific. Numerous dominant flightless mutants of *Act88F* have been described [24,28,44]. These dominant mutations were then classified on the basis of phenotype in the presence of two wild type alleles of *Act88F* as hypomorphic (including amorphic) or antimorphic. Hypomorphic alleles—flight ability restored in the presence of two wild type alleles in addition to the mutant allele—include premature stop codons at Trp80 and Gln122 [24,45], whereas more C-terminal truncations and many missense mutations were still flightless in the presence of two or more wild type alleles and therefore classified as antimorphic. Act88F and AeAct4 proteins are very similar; both 376 amino acids in length, only 17 amino acids differ from each other, they share 95% sequence similarity. On this basis we expected hypomorphic mutations in the region of *AeAct4* targeted to confer a dominant flightless phenotype, as for *Act88F* but female-specific since the expression of actin-coding *AeAct4* transcripts is restricted to females [26,27]. However, HDR knock-in of a fluorescent marker into the 5’ end of *AeAct4* coding region gave a recessive female-specific flightless phenotype instead. Consistent with this, CRISPR/Cas9 mutagenesis of *AeAct4* did not generate significant numbers of flightless G_0_ or G_1_ flightless individuals. This also suggests that a degree of mosaicism for *AeAct4* does not prevent flight, either because wild type muscle cells can compensate or because the syncytial nature of muscle cells means that enough *Act4* can be produced even if some nuclei are unable to do so.

Phenotypic analysis via dissection and examination of muscle fibers in *AeAct4*^hdr1^ heterozygote females showed no obvious visible defects, as seen in the TEM, which correlates with the observed flying phenotype. However, *AeAct4*^hdr1^ homozygote flightless females showed defects in myofibril structure in IFMs. The regular wild-type pattern of parallel filaments was disrupted, however the overall muscle structure of IFMs was maintained in homozygotes. Similar analysis of *Act88F* mutants showed that, perhaps surprisingly, neither *Act88F* nor the IFM-specific myosin heavy chain isoform are required for the development of muscle fibers [46].

Though the knock-out (amorph or strong hypomorph) phenotype is recessive, we additionally generated a series of dominant mutants. We observed a dominant female-specific flightless phenotype in *Cx*. *quinquefasciatus* carrying a CRISPR/Cas9-generated six-base pair deletion in *Act4*. We further showed that the same deletion gave a similar phenotype when replicated in *Aedes aegypti* by CRISPR/Cas9 oligo-directed mutagenesis. Although this does not prove that *CxAct4* is also haplosufficient, that hypothesis seems plausible. We additionally recovered a series of other dominant mutants, all being in-frame indels with net deletions of 3-36bp in the targeted region which are likely antimorphic. The fact that all of these mutations are in frame could be due to the fact that out of frame mutations are less likely to encode full length Act4-like proteins and hence be hypomorphic or amorphic meaning they would not have been detected in the mutant screen as performed since mutants would have been heterozygous.

A key motivation for analyzing the *Act4* mutant phenotype was to assess its relevance in genetic control. Loss of flight ability means that females cannot fly away from their larval habitats to seek a blood meal or to avoid predators. *Aedes aegypti* males—which do not bite or transmit disease—use female wingbeat frequency as a primary mate-recognition signal, therefore flightless females are also functionally sterile [20]. Flight is only relevant to adults, so the phenotype acts after any density-dependence effects acting on immature stages. For these reasons, dominant female-specific loss of flight has been proposed as a desirable engineered trait for genetic control [25,27]. Germline restriction of gene drive components is an important goal as unwanted somatic expression could lead to undesirable effects in fertility or viability which could prevent efficient gene drive. Therefore recessive female-specific lethal or sterile phenotypes are potentially useful for population suppression drives [2], especially where the gene function is not required in the germline or neighboring tissues. Further studies are required to assess any qualitative difference in flight ability in heterozygous females (which can fly by our assay) as well as the mating competitiveness of *AeAct4* mutant males to evaluate the potential of *AeAct4* in genetic control of mosquito populations as well as potential fertility effects in gene drive carriers.

Thus far, much attention has focused on genes involved in female fertility [19,47] but flight should provide another independent pathway to target, with no possibility of cross-resistance. *Act4* may also have desirable features in respect of cut-site resistance, a significant problem for CRISPR/Cas9-based homing drives [48,49]. A gene drive targeting a female-fertility gene (*AGAP007280*) in *An*. *gambiae* initially spread in experimental populations but then rapidly dropped out of the population [19,50]. Sequence analysis showed that this was due to accumulation of short in-frame deletions at the cut-site, presumably generated by end-joining repair of the CRISPR/Cas9-induced double-strand break. These deletions appeared to be functional, i.e. fertile, while preventing further cutting by CRISPR/Cas9 and so were strongly selected. In contrast, we found short indels in the targeted region of *Act4* to be dominant-negative. The targeting of alleles where mutations are likely to be deleterious has been suggested as a strategy to reduce resistance allele formation in homing drives [51], hence, *Act4* would be an interesting target in this sense.

The success of future genetic control strategies for the control of insect species is largely dependent on our growing understanding of their biology. In particular, these strategies will benefit from identifying those genes which are essential for reproduction or survival as the aim is to suppress, or if possible locally eradicate, vector populations. Since flight is a key part of mosquito reproduction and survival it is worth pursuing a better understanding of the specific genes and proteins involved.

## Supporting information

S1 Table *Cx*. *quinquefasciatus* and *Ae*. *aegypti Act4* sgRNA target sequences. Target sequences for the sgRNAs used in this study. Predicted cut sites are between the nucleotide numbers shown, counting the A of the ATG start codon as nucleotide 1.
(DOCX)

S2 Table *In vitro* cutting of *Cx*. *quinquefasciatus* and *Ae*. *aegypti Act4* sgRNAs. In vitro cleavage by sgRNAs was carried out at 37°C for indicated periods. Cutting efficiency of each sgRNA within each species is ranked and shown in brackets.
(DOCX)

S3 Table *Act4* mutation assay primers. Mutation assay primers to detect CRISPR/Cas9 mediated indels or HDR integrations in Act4.
(DOCX)

S4 Table ssODN and amplification primers used. *point where the 6 bases for the 156_161delTGCCTA deletion have been removed. The ssODN oligo consists of 60 nucleotides upstream and downstream of the deletion.
(DOCX)

S5 Table Plasmid sequences. Plasmid sequences corresponding to the 3xP3-mCherry-SV40 marker cassette used and the final AeAct4 HDR 3xP3-mCherry nDNA donor.
(DOCX)
