# Supplementary material for: Targeting female flight for genetic control of mosquitoes
Source: PLoS Negl Trop Dis. 2020 Dec 3;14(12):e0008876. doi: 10.1371/journal.pntd.0008876 (PMC7714197; doi:10.1371/journal.pntd.0008876)
Supplement: S1 Table — Target sequences for the sgRNAs used in this study. Predicted cut sites are between the nucleotide numbers shown, counting the A of the ATG start codon as nucleotide 1. (DOCX) [file pntd.0008876.s001.docx]

**S1 Table. *Cx. quinquefasciatus* and *Ae. aegypti*** ***Act4* sgRNA target sequences*.***

|  |  |  |  |  |
| --- | --- | --- | --- | --- |
| **Species** | ***Act4* sgRNA** | **Target Sequence (PAM sequence underlined)** | **+/- Strand** | **Cut site position (nt)** |
| *Cx. quinquefasciatus* | sgRNA 1 | GGAGCACTGGTCATTGACAACGG | - | 35/36 |
|  | sgRNA 2 | GGGTCAAAAGGATGCCTACGTGG | - | 160/161 |
|  | sgRNA 3 | GATGAAGCGCAATCGAAGAGAGG | - | 185/186 |
|  | sgRNA 4 | CGGTTGGACTTGGGATTCAAGGG | + | 333/334 |
| *Ae. aegypti* | sgRNA 1 | GGAGCACTAGTCATTGACAACGG | + | 35/36 |
|  | sgRNA 2 | GGTCAAAAAGATGCCTACGTGGT | + | 161/162 |
|  | sgRNA 3 | GTGCTCTATGGGATATTTCAGGG | - | 205/206 |
|  | sgRNA 4 | GTGGCGAGGGCGGCCGACAATGG | - | 106/107 |

Target sequences for the sgRNAs used in this study. Predicted cut sites are between the nucleotide numbers shown, counting the A of the ATG start codon as nucleotide 1.
