# Supplementary material for: Targeting female flight for genetic control of mosquitoes
Source: PLoS Negl Trop Dis. 2020 Dec 3;14(12):e0008876. doi: 10.1371/journal.pntd.0008876 (PMC7714197; doi:10.1371/journal.pntd.0008876)
Supplement: S2 Table — In vitro cleavage by sgRNAs was carried out at 37°C for indicated periods. Cutting efficiency of each sgRNA within each species is ranked and shown in brackets. (DOCX) [file pntd.0008876.s002.docx]

**S2 Table.** ***In vitro* cutting of *Cx. quinquefasciatus* and *Ae. aegypti*** ***Act4* sgRNAs*.***

|  |  | *In vitro* guiding for Cas9 cutting (37°C) | | |  |
| --- | --- | --- | --- | --- | --- |
| **Species** | ***Act4* sgRNA** | **15 min (rank within species)** | **2 hours (rank within species)** | **4 hours (rank within species)** |  |
| *Cx. quinquefasciatus* | sgRNA 1 | - | Very little (2^nd^) | Yes (3^rd^) |  |
|  | sgRNA 2 | - | Yes (1^st^) | Yes (1^st^) |  |
|  | sgRNA 3 | - | Very little (2^nd^) | Yes (2^nd^) |  |
|  | sgRNA 4 | - | Very little (2^nd^) | Yes (3^rd^) |  |
| *Ae. aegypti* | sgRNA 1 | Yes (2^nd^) | - | - |  |
|  | sgRNA 2 | Yes (1^st^) | - | - |  |
|  | sgRNA 3 | Yes (1^st^) | - | - |  |
|  | sgRNA 4 | No (3^rd^) | - | - |  |

*In vitro* cleavage by sgRNAs was carried out at 37 °C for indicated periods. Cutting efficiency of each sgRNA within each species is ranked and shown in brackets.

dsDNA donor.
