# Supplementary material for: Targeting female flight for genetic control of mosquitoes
Source: PLoS Negl Trop Dis. 2020 Dec 3;14(12):e0008876. doi: 10.1371/journal.pntd.0008876 (PMC7714197; doi:10.1371/journal.pntd.0008876)
Supplement: S3 Table — Mutation assay primers to detect CRISPR/Cas9 mediated indels or HDR integrations in Act4. (DOCX) [file pntd.0008876.s003.docx]

**S3 Table. *Act4* mutation assay primers.**

| Species | Primer Name | Primer Sequence | Description |
| --- | --- | --- | --- |
| *Cx. quinquefasciatus AeAct4* Primers | LA350 | GACGCTCTCTGCCGCAGACTGTACT | Amplicon Primers |
|  | LA351 | AGTCTCGTGGACACCGGTAGCTTCC |  |
|  | LA388 | CGTCCGAGTTGTTTGTGGATG | Sequencing Primers |
|  | LA205 | CGCTCGGTCAGGATCTTCAT |  |
| *Ae. aegypti AeAct4* Primers | LA587 | AACGAGCCCTGTACCTATTGAT | Amplicon Primers |
|  | LA130 | GTACAGGGACAGAACAGCTTGGAT |  |
|  | LA129 | TGCCACATGAACAATAACACCAATA | Sequencing Primers |
|  | LA130 | GTACAGGGACAGAACAGCTTGGAT |  |

Mutation assay primers to detect CRISPR/Cas9 mediated indels or HDR integrations in *Act4*.
