# Supplementary material for: Targeting female flight for genetic control of mosquitoes
Source: PLoS Negl Trop Dis. 2020 Dec 3;14(12):e0008876. doi: 10.1371/journal.pntd.0008876 (PMC7714197; doi:10.1371/journal.pntd.0008876)
Supplement: S4 Table — *point where the 6 bases for the 156_161delTGCCTA deletion have been removed. The ssODN oligo consists of 60 nucleotides upstream and downstream of the deletion. (DOCX) [file pntd.0008876.s004.docx]

**S4 Table. ssODN and amplification primers used.**

| Oligo Name | Description | Primer Sequence | Size (nt) | +/- Strand |
| --- | --- | --- | --- | --- |
| LA1422 | ssODN for 156_161delTGCCTA AeAct4 mutagenesis | TGCTCTATGGGATATTTCAGGGTGAGGATACCTCGCTTGGATTGGGCTTCATCACCGACG*TCTTTTTGACCCATACCGACCATCACACCCTGGTGGCGAGGGCGGCCGACAATGGACGGG | 120 | - |
| LA706 | 5’*Act4* Homology Arm + BsaI site amplified from *Ae. aegypti* gDNA | GGTCTCAGGCCGCACTTCCGAGTATAAAACCCCGGT | 36 | + |
| LA709 |  | GGTCTCTGCAGTCAATGACTAGTGCTCCAGCATCATC | 37 | - |
| LA712 | 3’*Act4* Homology Arm + BsaI site amplified from *Ae. aegypti* gDNA | GGTCTCAGCTCGCTAGCAATATCCCATAGAGCACGGTATCATCAC | 45 | + |
| LA707 |  | GGTCTCACATGACCAAGAACAGTTTAGAATCGAGACG | 37 | - |
| LA710 | attP50-SV40-mCherry-3xP3 + BsaI amplified from AGG1069 plasmid | GGTCTCACTGCCGAATTGCTTCGGCGCCAAGTAGTGCC | 38 | + |
| LA711 |  | GGTCTCAGAGCGGCGCGCCGCCCGGGGATCTAATTCAATTAGAG | 44 | - |

*point where the 6 bases for the 156_161delTGCCTA deletion have been removed. The ssODN oligo consists of 60 nucleotides upstream and downstream of the deletion.
