# Supplementary material for: Targeting female flight for genetic control of mosquitoes
Source: PLoS Negl Trop Dis. 2020 Dec 3;14(12):e0008876. doi: 10.1371/journal.pntd.0008876 (PMC7714197; doi:10.1371/journal.pntd.0008876)
Supplement: S5 Table — Plasmid sequences corresponding to the 3xP3-mCherry-SV40 marker cassette used and the final AeAct4 HDR 3xP3-mCherry nDNA donor. (DOCX) [file pntd.0008876.s005.docx]

**S5 Table. Plasmid sequences.**

| Plasmid Name | Nucleotide Sequence | Size (bp) |
| --- | --- | --- |
| AGG1069 | tctagctggtggcacttttcggggaaatgtgcgcggaacccctatttgtttatttttctaaatacattcaaatatgtatccgctcatgagacaataaccctgataaatgcttcaataatattgaaaaaggaagagtatgagtattcaacatttccgtgtcgcccttattcccttttttgcggcattttgccttcctgtttttgctcacccagaaacgctggtgaaagtaaaagatgctgaagatcagttgggtgcacgagtgggttacatcgaactggatctcaacagcggtaagatccttgagagttttcgccccgaagaacgttttccaatgatgagcacttttaaagttctgctatgtggcgcggtattatcccgtattgacgccgggcaagagcaactcggtcgccgcatacactattctcagaatgacttggttgagtactcaccagtcacagaaaagcatcttacggatggcatgacagtaagagaattatgcagtgctgccataaccatgagtgataacactgcggccaacttacttctgacaacgatcggaggaccgaaggagctaaccgcttttttgcacaacatgggggatcatgtaactcgccttgatcgttgggaaccggagctgaatgaagccataccaaacgacgagcgtgacaccacgatgcctgtagcaatggcaacaacgttgcgcaaactattaactggcgaactacttactctagcttcccggcaacaattaatagactggatggaggcggataaagttgcaggaccacttctgcgctcggcccttccggctggctggtttattgctgataaatctggagccggtgagcgtgggtctcgcggtatcattgcagcactggggccagatggtaagccctcccgtatcgtagttatctacacgacggggagtcaggcaactatggatgaacgaaatagacagatcgctgagataggtgcctcactgattaagcattggtaactgtcagaccaagtttactcatatatactttagattgatttaaaacttcatttttaatttaaaaggatctaggtgaagatcctttttgataatctcatgaccaaaatcccttaacgtgagttttcgttccactgagcgtcagaccccgtagaaaagatcaaaggatcttcttgagatcctttttttctgcgcgtaatctgctgcttgcaaacaaaaaaaccaccgctaccagcggtggtttgtttgccggatcaagagctaccaactctttttccgaaggtaactggcttcagcagagcgcagataccaaatactgttcttctagtgtagccgtagttaggccaccacttcaagaactctgtagcaccgcctacatacctcgctctgctaatcctgttaccagtggctgctgccagtggcgataagtcgtgtcttaccgggttggactcaagacgatagttaccggataaggcgcagcggtcgggctgaacggggggttcgtgcacacagcccagcttggagcgaacgacctacaccgaactgagatacctacagcgtgagctatgagaaagcgccacgcttcccgaagggagaaaggcggacaggtatccggtaagcggcagggtcggaacaggagagcgcacgagggagcttccagggggaaacgcctggtatctttatagtcctgtcgggtttcgccacctctgacttgagcgtcgatttttgtgatgctcgtcaggggggcggagcctatggaaaaacgccagcaacgcggcctttttacggttcctggccttttgctggccttttgctcacatgttctttcctgcgttatcccctgattctgtggataaccgtattaccgcctttgagtgagctgataccgctcgccgcagccgaacgaccgagcgcagcgagtcagtgagcgaggaagcggaagagcgcccaatacgcaaaccgcctctccccgcgcgttggccgattcattaatgcagctggcacgacaggtttcccgactggaaagcgggcagtgagcgcaacgcaattaatgtgagttagctcactcattaggcaccccaggctttacactttatgcttccggctcgtatgttgtgtggaattgtgagcggataacaatttcacacaggaaacagctatgaccatgattacgccaagctcgctagattaaccctagaaagatagtctgcgtaaaattgacgcatgcattcttgaaatattgctctctctttctaaatagcgcgaatccgtcgctgtgcatttaggacatctcagtcgccgcttggagctcccgtgaggcgtgcttgtcaatgcggtaagtgtcactgattttgaactataacgaccgcgtgagtcaaaatgacgcatgattatcttttacgtgacttttaagatttaactcatacgataattatattgttatttcatgttctacttacgtgataacttattatatatatattttcttgttatagatatcgctagcatgaattcgcccggggatctaattcaattagagactaattcaattagagctaattcaattaggatccaagcttatcgatttcgaaccctcgaccgccggagtataaatagaggcgcttcgtctacggagcgacaattcaattcaaacaagcaaagtgaacacgtcgctaagcgaaagctaagcaaataaacaagcgcagctgaacaagctaaacaatcggggtaccgctagagtcgaatggtgagcaagggcgaggaggataacatggccatcatcaaggagttcatgcgcttcaaggtgcacatggagggctccgtgaacggccacgagttcgagatcgagggcgagggcgagggccgcccctacgagggcacccagaccgccaagctgaaggtgaccaagggtggccccctgcccttcgcctgggacatcctgtcccctcagttcatgtacggctccaaggcctacgtgaagcaccccgccgacatccccgactacttgaagctgtccttccccgagggcttcaagtgggagcgcgtgatgaacttcgaggacggcggcgtggtgaccgtgacccaggactcctccctgcaggacggcgagttcatctacaaggtgaagctgcgcggcaccaacttcccctccgacggccccgtaatgcagaagaagaccatgggctgggaggcctcctccgagcggatgtaccccgaggacggcgccctgaagggcgagatcaagcagaggctgaagctgaaggacggcggccactacgacgctgaggtcaagaccacctacaaggccaagaagcccgtgcagctgcccggcgcctacaacgtcaacatcaagttggacatcacctcccacaacgaggactacaccatcgtggaacagtacgaacgcgccgagggccgccactccaccggcggcatggacgagctgtacaagtaatgatcataatcagccataccacatttgtagaggttttacttgctttaaaaaacctcccacacctccccctgaacctgaaacataaaatgaatgcaattgttgttgttaacttgtttattgcagcttataatggttacaaataaagcaatagcatcacaaatttcacaaataaagcatttttttcactgcattctagttgtggtttgtccaaactcatcaatgtatcttactcgacctacgcccccaactgagagaactcaaaggttaccccagttggggcactacttggcgccgaagcaattcgataaaagttttgttactttatagaagaaattttgagtttttgtttttttttaataaataaataaacataaataaattgtttgttgaatttattattagtatgtaagtgtaaatataataaaacttaatatctattcaaattaataaataaacctcgatatacagaccgataaaacacatgcgtcaattttacgcatgattatctttaacgtacgtcacaatatgattatctttctagggttaa | 3996 |
| AGG1070 | catggaaaatcgatgttcttcttttattctctcaagattttcaggctgtatattaaaacttatattaagaactatgctaaccacctcatcaggaaccgttgtaggtggcgtgggttttcttggcaatcgactctcatgaaaactacgagctaaatattcaatatgttcctcttgaccaactttattctgcattttttttgaacgaggtttagagcaagcttcaggaaactgagacaggaattttattaaaaatttaaattttgaagaaagttcagggttaatagcatccattttttgctttgcaagttcctcagcattcttaacaaaagacgtctcttttgacatgtttaaagtttaaacctcctgtgtgaaattgttatccgctcacaattccacacattatacgagccggaagcataaagtgtaaagcctggggtgcctaatgagtgagctaactcacattaattgcgttgcgctcactgccaattgctttccagtcgggaaacctgtcgtgccagctgcattaatgaatcggccaacgcgcggggagaggcggtttgcgtattgggcgctcttccgcttcctcgctcactgactcgctgcgctcggtcgttcggctgcggcgagcggtatcagctcactcaaaggcggtaatacggttatccacagaatcaggggataacgcaggaaagaacatgtgagcaaaaggccagcaaaaggccaggaaccgtaaaaaggccgcgttgctggcgtttttccataggctccgcccccctgacgagcatcacaaaaatcgacgctcaagtcagaggtggcgaaacccgacaggactataaagataccaggcgtttccccctggaagctccctcgtgcgctctcctgttccgaccctgccgcttaccggatacctgtccgcctttctcccttcgggaagcgtggcgctttctcatagctcacgctgtaggtatctcagttcggtgtaggtcgttcgctccaagctgggctgtgtgcacgaaccccccgttcagcccgaccgctgcgccttatccggtaactatcgtcttgagtccaacccggtaagacacgacttatcgccactggcagcagccactggtaacaggattagcagagcgaggtatgtaggcggtgctacagagttcttgaagtggtggcctaactacggctacactagaaggacagtatttggtatctgcgctctgctgaagccagttaccttcggaaaaagagttggtagctcttgatccggcaaacaaaccaccgctggtagcggtggtttttttgtttgcaagcagcagattacgcgcagaaaaaaaggatctcaagaagatcctttgatcttttctacggggtctgacgctcagtggaacgaaaactcacgttaagggattttggtcatgagattatcaaaaaggatcttcacctagatccttttaaattaaaaatgaagttttaaatcaatctaaagtatatatgagtaaacttggtctgacagttaccaatgcttaatcagtgaggcacctatctcagcgatctgtctatttcgttcatccatagttgcctgactccccgtcgtgtagataactacgatacgggagggcttaccatctggccccagtgctgcaatgataccgcgagacccacgctcaccggctccagatttatcagcaataaaccagccagccggaagggccgagcgcagaagtggtcctgcaactttatccgcctccatccagtctattaattgttgccgggaagctagagtaagtagttcgccagttaatagtttgcgcaacgttgttgccattgctacaggcatcgtggtgtcacgctcgtcgtttggtatggcttcattcagctccggttcccaacgatcaaggcgagttacatgatcccccatgttgtgcaaaaaagcggttagctccttcggtcctccgatcgttgtcagaagtaagttggccgcagtgttatcactcatggttatggcagcactgcataattctcttactgtcatgccatccgtaagatgcttttctgtgactggtgagtactcaaccaagtcattctgagaatagtgtatgcggcgaccgagttgctcttgcccggcgtcaatacgggataataccgcgccacatagcagaactttaaaagtgctcatcattggaaaacgttcttcggggcgaaaactctcaaggatcttaccgctgttgagatccagttcgatgtaacccactcgtgcacccaactgatcttcagcatcttttactttcaccagcgtttctgggtgagcaaaaacaggaaggcaaaatgccgcaaaaaagggaataagggcgacacggaaatgttgaatactcatactcttcctttttcaatattattgaagcatttatcagggttattgtctcatgagcggatacatatttgaatgtatttagaaaaataaacaaataggggttccgcgcacatttccccgaaaagtgccacctgacgtctaagaaaccattattatcatgacattaacctataaaaataggcgtatcacgaggccgcccctgcagccgaattatattatttttgccaaataatttttaacaaaagctctgaagtcttcttcatttaaattcttagatgatacttcatctggaaaattgtcccaattagtagcatcacgctgtgagtaagttctaaaccatttttttattgttgtattatctctaatcttactactcgatgagttttcggtattatctctatttttaacttggagcaggttccattcattgtttttttcatcatagtgaataaaatcaactgctttaacacttgtgcctgaacaccatatccatccggcgtaatacgactcactatagggagagcggccgcacttccgagtataaaaccccggtaaacccaaggaatcactcacaatcggattttgacgctcgctctggtacagttcgatacggtctagtgaaaccgaggataacgacgaaggtttttccccattgatccaggtcggtgtttatgattggtggaaaaagagctcgagaaaagttccatcgaagccgttggaaatgtgccgtcttcctgtgacgtcttgtggatccagttccttgttcacgtctggtgatcgtgtaaaatgtgctgtcttgtggcgtcatatgtgttccagatccagtgattacgatccgatgtgatgttgatcccttgtgaacgtcttatcctgttccgtgtgcaccatgcataatgtcgtattacgtaagttctgaagtgaaacagaagagtgaattgaaagtttttttattcaacatcaacctaaatatggactttactttccaagaaaattatgcctgatcaactgtggatagttacaaaaaaaaaaggtttattaattaaattttatgattacataatgtgttgaaaagaacaactgaaattttagaagaagatcttttcgtgcatcaggctttgccaattaattgatgataaattatcatagcaaattaacgtagagactaaaaggtatatcgtcaaatagggcttcttttgacactattttggcattcttgctctttgagaacttgcaaccctaaaatgggatcttcatcagcctagtggttagattcagcagctacaaagcaaaaccatgctgaagggttcgattcccggtcgtttcaggatcttttcgtaattgaaatatccttgactaccctaagtatcattgtgcttgccatttacgaatatacatattacgatatacgaatgagaaaatgacaactttggaaaataaagctctcaatgtttcaataagaaataaatactacatcagtattgaaggctaataacaattacagattagaacctttaaacatcatttctgcaacaggctggataaagtacagttggaggattaaattatgcgattttgcaattttttccgattaaattcatatttattcctggtttggtttttacaaaaaatatttttacatgacgtttgaccccgattccctcaactttgattgttatatttttttttggacaggttgagtttgtgggttttttcctagtgttgctttgctttatgggctctggttatttaaaattaaaatttgacaatcttactacacactccgaaaaaatcatgcgattttacgtcttttggatgcacataaaagaagcgagccaaatgaggtgaatttgtgtcacattttaaatacgatggtgtctgattcgggaaatgtcaatgatagtgtcattcaatcataatgtgaattacgtccgcagtaattttcattatttttaagagtgtactactatttacactacaaaaattttgataccccaggggggaacgaggtcccggatgtccagctggccagattgttggcaacgagccctgtacctattgatcgagtcaccaaagcactcctcaagtgttttaatctcgaccagacggtggacctcggttgttctcattctcggagggcgatttcgcaatcattagtaccaaccacatgtcgaagtcgggagatgttataaaattataaccaattattcaaaaaatgacatcattcaatttgaacaaacgttcgatagaaattatatatgatttcacatgatattaaactacgaagaaaattttacataaggaagtggtataaaacgtaatatgcttaataaaaactttaacccttttgggaggataatattcagaagttctgattcagaaccatctctcatgttatgttcgttttttgttgcttgtcctttatatgccacatgaacaataacaccaatatctatcccatttccaggacctaacggaccttgaagcggcgccaaaatgtgtgacgatgatgctggagcactagtcattgactgccgaattgcttcggcgccaagtagtgccccaactggggtaacctttgagttctctcagttgggggcgtaggtcgagtaagatacattgatgagtttggacaaaccacaactagaatgcagtgaaaaaaatgctttatttgtgaaatttgtgatgctattgctttatttgtaaccattataagctgcaataaacaagttaacaacaacaattgcattcattttatgtttcaggttcagggggaggtgtgggaggttttttaaagcaagtaaaacctctacaaatgtggtatggctgattatgatcattacttgtacagctcgtccatgccgccggtggagtggcggccctcggcgcgttcgtactgttccacgatggtgtagtcctcgttgtgggaggtgatgtccaacttgatgttgacgttgtaggcgccgggcagctgcacgggcttcttggccttgtaggtggtcttgacctcagcgtcgtagtggccgccgtccttcagcttcagcctctgcttgatctcgcccttcagggcgccgtcctcggggtacatccgctcggaggaggcctcccagcccatggtcttcttctgcattacggggccgtcggaggggaagttggtgccgcgcagcttcaccttgtagatgaactcgccgtcctgcagggaggagtcctgggtcacggtcaccacgccgccgtcctcgaagttcatcacgcgctcccacttgaagccctcggggaaggacagcttcaagtagtcggggatgtcggcggggtgcttcacgtaggccttggagccgtacatgaactgaggggacaggatgtcccaggcgaagggcagggggccacccttggtcaccttcagcttggcggtctgggtgccctcgtaggggcggccctcgccctcgccctcgatctcgaactcgtggccgttcacggagccctccatgtgcaccttgaagcgcatgaactccttgatgatggccatgttatcctcctcgcccttgctcaccattcgactctagcggtaccccgattgtttagcttgttcagctgcgcttgtttatttgcttagctttcgcttagcgacgtgttcactttgcttgtttgaattgaattgtcgctccgtagacgaagcgcctctatttatactccggcggtcgagggttcgaaatcgataagcttggatcctaattgaattagctctaattgaattagtctctaattgaattagatccccgggcggcgcgccgctcgctagcaatatcccatagagcacggtatcatcaccaactgggatgatatggagaagatttggcatcacaccttctacaacgagttgcgagtagctcctgaagaacatccagtattgctgactgaggctcccttgaatccaaagtccaatcgcgagaagatgactcagatcatgtttgaaacattcgcttcgccagctgtgtatgttgccatccaagctgttctgtccctgtacgcctccggtcgtactactggtattgttctggattccggagatggtgtctcccataccgtcccaatctacgaaggttatgctctgccacatgccatcctccgtatggatttggctggtcgtgatctgaccgactacctgatgaagatcttgaccgaacgtggatactctttcaccaccaccgctgaacgtgaaatcgttcgtgacatcaaggagaagctgtgctacgtcgctctggacttcgagcaggaaatgcaagccgctgccgctacgtcttcatccgagaagtcttatgaacttcccgatggccaagtcatcacaatcggcaacgagcgtttccgtgctccagaagcccttttccagccatccttcctgggaatggaatcaactggcattcatgaaacggtctacaactcgatcatgcgttgcgatgtcgacatccgcaaggatctctatgctaacagcgtcttgtctggtggtaccaccatgtacccaggtatttcttatatttaaccactcaactctgcatcatactcaaaacctccctctattacaggtattgctgatcgtatgcagaaggaaatcacttccctggctccatccaccatcaagatcaagatcattgccccaccggaacgtaaatactccgtctggatcggtggatccatcctggcctcgctgtctaccttccaagctatgtggatctccaagcaggaatacgacgaaggtggcccaggaattgtccaccgcaagtgcttctaagccgatcccgattgtactgattaccataagcgacattgccagtgaaagcgacaacagcagcatcaaagtacatttgtcatactgattcggctactaccaccatccggaatcagcttgcatcgaacatcaaatcacgttattcaatgtatctgtcatccagctcagacaagtcggagcttttccagtcgcgaaaatctgcgactccagcggaaagcaccgaaccacagagaggactcgtatgaaagccagggaagaaaccatcattcaccttgcagcaaataggaaaaaaaaacggacatcttcaacaaacaaaagcccatgcgctaacttggtttaggagtttagtgtgacaccatgaccccgctgatgatctttacttagcaccataaccacctttatgcgttcgttcatccaaaatctacaggatatcactgcagccgcgagaagaactcgtgaaccatcctgttttcttttttattatattcttacttttaacttcaaattattttcagtaataaaacgtctcaaaataataagttcataatgagtttaattttacggaataagaacaaccatttaagttattaaatccttagatttaatggaattagattgattatatggaacccagacttggtaaaaaataaactccacgttataattctttctgagacttaaaattctttcgggaaagctgggagcaattctcgctaaggcgtcgtccacaaattatgaaacgctttaattacgtgacggagtaggctcaagcgtacgaatcatacaaaaataatacaaatttttcatataaaaagcgttacgaagggggaggtggtcgaaaattgacaattgaccggcccaatctttgacattagttttctttaataattaaaattatgcttgatttaaaattcatctcgagtcatctctgaattcggttgttcaattgcatgggtccttcgttagttataattaaaaacgtgtttgaggacgtctcgattctaaactgttcttggt | 8165 |

Plasmid sequences corresponding to the 3xP3-mCherry-SV40 marker cassette used and the final AeAct4 HDR 3xP3-mCherry dsDNA donor.
